# Myocardial systolic strain assessed by cardiovascular magnetic resonance relates to subclinical atherosclerosis in healthy young adults

**DOI:** 10.1186/1532-429X-13-S1-O95

**Published:** 2011-02-02

**Authors:** Adam J Lewandowski, Merzaka Lazdam, Jonathan Diesch, Jane M Francis, Daniel Augustine, Rajarshi Banerjee, Joseph J Suttie, Stefan Neubauer, Paul Leeson

**Affiliations:** 1 University of Oxford, Oxford, UK

## Background

Cardiovascular magnetic resonance (CMR) allows quantification of early changes in systolic myocardial function. In the elderly, reduced left ventricular systolic function is related to elevated carotid intima media thickness (IMT), a well-established marker of atherosclerosis. We therefore sought to determine if peak myocardial systolic strain was also related to subclinical variation in carotid IMT and cardiovascular risk factors in young adults.

## Methods

We recruited 68 individuals (39 females, 29 males) with a mean age of 28.29±5.45 (mean±SD). Peak mid-ventricular myocardial circumferential systolic strain and left ventricular mass adjusted for body surface area (LVM) were assessed by CMR. Carotid IMT was measured as a marker of subclinical atherosclerosis using ultrasound. Demographic and anthropometric characteristics were measured as well as metabolic parameters and peripheral blood pressure.

## Results

Individuals with reduced peak myocardial circumferential systolic strain had higher carotid IMT (*r*=0.406, *P*=0.001). Elevated total cholesterol and waist to hip ratio were both significantly associated with reduced myocardial strain. Increased LVM, peripheral systolic blood pressure, pulse pressure, glucose, triglycerides, age, BMI and WHR, as well as reduced HDL, were all significantly associated with increased carotid IMT (*P*<0.01). Males also had higher carotid IMT than females (mean±SD = 0.55±0.071mm vs 0.48±0.059mm, *P*=0.001). However, the association between carotid IMT and peak myocardial circumferential systolic strain was independent of gender and LVM.

## Conclusions

We show for the first time associations between subclinical cardiac function and subclinical atherosclerosis in young adults. This study demonstrates the ability of CMR to detect subtle changes in cardiac function relevant to cardiovascular disease development in young people.

